# Spatial distribution and environmental factors associated to phlebotomine fauna in a border area of transmission of visceral leishmaniasis in Mato Grosso do Sul, Brazil

**DOI:** 10.1186/1756-3305-7-260

**Published:** 2014-06-04

**Authors:** Ana Rachel Oliveira de Andrade, Baldomero Antonio Kato da Silva, Geucira Cristaldo, Sonia Maria Oliveira de Andrade, Antonio Conceição Paranhos Filho, Alisson Ribeiro, Mirella Ferreira da Cunha Santos, Renato Andreotti

**Affiliations:** 1 Post Graduate Program in Infectious and Parasitary Diseases, Federal University of Mato Grosso do Sul, Campus Universitário, s/n, Caixa Postal 549, CEP 79070-900 Campo Grande, Mato Grosso do Sul, Brasil; 2 Center of Health Sciences, Federal University of Piauí, Campus Universitário Ministro Reis Velloso, Av. São Sebastião 2819, Bairro São Benedito, CEP 64202-020 Parnaíba, Piauí, Brasil; 3 Human Parasitology Laboratory, Federal University of Mato Grosso do Sul, Campus Universitário, s/n, Caixa Postal 549, CEP 79070-900 Campo Grande, Mato Grosso do Sul, Brasil; 4 Post Graduate Program in Health and Development of the Central West Region, Federal University of Mato Grosso do Sul, Campus Universitário, s/n, Caixa Postal 549, CEP 79070-900 Campo Grande, Mato Grosso do Sul, Brasil; 5 Geotechnology Laboratory, Federal University of Mato Grosso do Sul, Campus Universitário, s/n, Caixa Postal 549, CEP 79070-900 Campo Grande, Mato Grosso do Sul, Brasil; 6 Embrapa Beef Cattle, Campo Grande, BR 262 km 4 - Caixa Postal 154, CEP 79002-970 Campo Grande, Mato Grosso do Sul Brasil

**Keywords:** Border area, Geotechnology, Phlebotomines

## Abstract

**Background:**

Mato Grosso do Sul has been undergoing a process of urbanization which results in loss of native vegetation. This withdrawal makes vectors of man and domestic animals closer, causing changes in the epidemiology of diseases such as American Visceral Leishmaniasis. The aim of the study was to evaluate the phlebotomine fauna and environmental issues related to the transmission of AVL in Ponta Porã, Mato Grosso do Sul, between 2009 and 2010.

**Methods:**

Vegetation of the urban area was evaluated by Normalized Difference Vegetation Index (NDVI), Normalized Difference Water Index (NDWI) and Soil Adjusted Vegetation Index (SAVI).

**Results:**

The results showed that the phlebotomine fauna of the city consists of five species, especially *Lutzomyia longipalpis* (Lutz and Neiva, 1912), the vector of *Leishmania (Leishmania) infantum.* Predominance of males was observed. The insects were captured in greater quantity in the intradomicile. *Lu. longipalpis* was the most frequent and abundant species, present throughout the year, with a peak population after the rainy season. Vectors can be found in high amounts in forest and disturbed environments.

**Conclusions:**

The finding of *Lu. longipalpis* in regions with little vegetation and humidity suggests that the species is adapted to different sorts of environmental conditions, demonstrating its close association with man and the environment it inhabits. The tourist feature of Ponta Porã reinforces its epidemiological importance as a vulnerable city. The geographical location, bordering Paraguay through dry border, makes possible the existence of a corridor of vectors and infected dogs between the two countries.

## Background

Over the years, studies on the phlebotomine fauna have been carried out in countries bordering the state of Mato Grosso do Sul [1,2]. The growth in number of vectors, humans and dogs infected in the State leads to the development of studies in the cities in order to observe the epidemiological pattern of the disease [3-5].

The presence of sandflies is associated with disturbed or forested environments [6], so every time that factors such as vegetation, humidity and temperature of a site are analyzed, indirectly the development conditions of the vector are evaluated.

Antonialli *et al.*[7] used spatial analysis to demonstrate the evolutionary patterns of American visceral leishmaniasis in Mato Grosso do Sul state. Oliveira *et al.*[5] evaluated the influence of environmental variables by Normalized Difference Vegetation Index (NDVI) by correlating the distribution of *Lu. Longipalpis*, canine and human cases of AVL in Campo Grande. These studies show how geotechonologies can be used as an auxiliary method for detecting ecological changes and delineating risk areas, aiming to support the development of strategies for the control of leishmaniases.

The objective of this study was to evaluate the sandfly fauna and environmental issues related to the transmission of AVL in Ponta Porã, Mato Grosso do Sul, between the years 2009 and 2010.

## Methods

### Study area

The city of Ponta Porã is located in the south of Mato Grosso do Sul, in the microregion of Dourados, 326 km away from the capital, Campo Grande. The average altitude of Ponta Porã (22° 53′ 10″ S 55° 42′ 32″ W) is 655 m above sea level [8].

Its current population is 79,173 inhabitants, of whom 89% live in urban areas and 11% in rural areas, having a population density of 14.61 (inhabitants/km^2^) [8].

As far as vegetation is concerned, there is predominance of grasslands as a characteristic of the city, formed by large areas of low grass, forming the famous natural pastures. Tropical highland climate with dry winters predominates in the area, with annual average temperature of 20.6°C and monthly average temperature of 23°C.

The hottest month is February with an average temperature of 23.6°C, and the coldest month is July, with a mean of 16.4°C. The average annual rainfall is 1.660 mm, with periods of wetter summers than the winter. The wettest month is November with an average of 212 mm, while the driest is July, with an average of 55 mm. It is under the influence of the Prata River Basin, being serviced primarily by Dourados River [8].

### Entomological data

The phlebotomines were captured with CDC automatic light traps fortnightly installed at seven sites in Ponta Porã, both intradomicile and in the peridomiciles, totaling 14 ecotopes. Collections were carried out in the period between April 2009 and March 2010, from 6 pm to 6 am, disregarding the summer time change. The sites were chosen so they could comprise the whole urban area, considering the healthcare regions already defined for the municipality actions.

In one of the capture sites a white Shannon trap was installed on a monthly basis to check which sandfly species existed and their anthropophily.

The phlebotomines captured with CDC were separated from other insects; males and females were placed on Petri dishes and taken to the Parasitology Laboratory of the Federal University of Mato Grosso do Sul, where they were clarified according to Foratinni’s methodology and then arranged on plates [9].

The structures of the head, thorax and abdomen were used for species identification. In specific levels the emphasis was given to the classification proposed by Galati [10,11] and the abbreviation proposed by Marcondes [12].

### Environmental analysis

As cartographic basis we have used an image taken from Landsat 5 TM, orbit-point 225/076, on August 18^th^ 2010, freely available on the National Institute for Space Research’s website [13].

For georeferencing, a previously corrected orthorectified image of Landsat 7 ETM+, orbit-point 225/076 of April 8^th^ 2000, was used. The projection and datum used were respectively UTM (zone 21) and WGS84 [14].

The atmospheric correction of the image was made in *software* Geomatica, through algorithm atcor2 [15]. On the corrected image, Normalized Difference Vegetation Index (NDVI), Normalized Difference Water Index (NDWI) and Soil Adjusted Vegetation Index (SAVI) were calculated [16].

NDVI allows the characterization of biophysical parameters of vegetation, such as biomass/density of vegetation and its value is normalized to the range of -1.00 to +1.00. These values represent an indirect measurement of phytomass, indicating values of matter and energy present in the sampled area [17].

NDVI is calculated by the following equation:

NDVI=NI-RNIR+R

where:

NIR: reflectance of vegetation in the near infrared band.

R: Reflectance of vegetation in the red band.

Concerning the limitations caused by the noise of background soil on NDVI, SAVI was used, ranging from + 0.00 to 1.00, an index proposed by Huete [18], that allows adjustment of the background soil (L).

$$\mathrm{SAVI}=\left(1+L\right)\mathrm{NIR}-\mathrm{RNIR}+R+L$$

NDWI reflects the water content in vegetation (*Vegetation Water Index* - VWC). VWC is an important parameter in studies of vegetation and soil moisture with values ranging from -1.00 to +1.00. Gao [19] developed NDWI for determining VWC, and NDWI is given by:

NDWI=NIR-SWIRNIR+SWIR,

where NIR is the reflectance of vegetation in the near infrared band, and SWIR is the reflectance of vegetation in the mid-infrared band.

### Statistical analysis

The proportion of genders was compared with the Z-test and p-value to test the hypothesis. The same test was used to compare the capture condition (intra or peridomicile). Correlations between number of specimens and georeferenced data, canine cases and georeferenced data, number of exemplars caught and number of canine cases, and number of *Lu. longipalpis* and number of human cases were realized with Spearman’s Correlation Coefficient. In all cases, the p-value used to demonstrate statistical significance was 0.05. All statistical analysis was calculated using Bioestat 5.0.

### Ethics statement

The study received approval from the local Animal Experimentation Ethics Committee (CEUA/UFMS) under protocol number 206/2009. Capture sites were located in private areas, and all owners gave permission to conduct the study in these sites. The field studies did not involve endangered/protected species or protected areas.

## Results

A total of 707 insects were captured by automatic modified light traps (565 males and 142 females), in a ratio of males to females of 3,97:1 (p <0.0001). Of the speciemens collected, 435 (61.5%) were captured in the intradomicile and 272 (38.5%) in the peridomicile (p < 0.0001), 79.92% males and 20.08% females, belonging to three sub-tribes, five genera and five species: Lutzomyiina - *Lutzomyia longipalpis* (Lutz & Neiva, 1912) and *Evandromyia cortelezzii* (Brethes, 1923); Bumptomyiina - *Brumptomyia brumpti* (Larousse, 1920); Psychodopygina - *Nyssomyia whitmani* (Antunes & Coutinho, 1939) and *Psathyromyia shannoni* (Dyar, 1929) (Table 1).

**Table 1:** Phlebotomines captured fortnightly with CDC automatic light traps, by species, sex and site of capture

**Phlebotomines**	**Site 01**		**Site 02**		**Site 03**		**Site 04**		**Site 05**		**Site 06**		**Site 07**		**Total**			
	**M**	**F**	**M**	**F**	**M**	**F**	**M**	**F**	**M**	**F**	**M**	**F**	**M**	**F**	**M**	**F**	**MF**	**%**
*Br. brumpti*	-	-	-	-	-	01	-	-	-	-	-	-	-	-	-	01	01	0.14
*Ev. cortelezzii*	-	-	-	02	-	03	-	-	-	-	-	-	-	-	-	05	05	0.71
*Lu. longipalpis*	03	-	141	24	195	46	216	56	-	03	02	-	-	-	557	129	686	97.03
*Ny. whitmani*	-	-	03	03	01	01	-	-	-	-	-	-	-	-	04	04	08	1.13
*Ps. shannoni*	-	-	-	-	04	03	-	-	-	-	-	-	-	-	04	03	07	0.99
**TOTAL**	**03**	**-**	**144**	**29**	**200**	**54**	**216**	**56**	**-**	**03**	**02**	**-**	**-**	**-**	**565**	**142**	**707**	**100**

M: male; F: female.

*Lu. longipalpis* was the most frequent species, representing 686 (97,03%) of specimens collected. It was also the only one found in all capture sites, occurring in only eight of 14 ecotopes of Ponta Porã.

Between April 2009 and March 2010, six cases of AVL and 50 cases of canine visceral leishmaniasis (CVL) were reported in Ponta Porã. The secondary data on the occurrence of human and canine cases and canines in the study areas, were obtained through the Department of State Health Mato Grosso do Sul and the Center for Zoonosis Control Ponta Porã (Table 2). These cases occurred in only three of the seven studied areas. No correlation was observed between the number of specimens caught and the number of canine cases (r = 0.6841, p = 0.0901), nor between the number of *Lu. longipalpis* and the number of human cases in the capture sites (r = 0.6761, p = 0.0954).

**Table 2:** NDVI, NDWI, SAVI, AVL cases and CVL cases in all sites of capture

	**NDVI**	**NDWI**	**SAVI**	**AVL cases**	**CVL cases**
Site 01	0.406861	-0.105564	0.608469	-	03
Site 02	0.375183	-0.047968	0.560971	01	19
Site 03	0.269149	-0.122483	0.402167	-	-
Site 04	0.585989	0.100511	0.875453	05	28
Site 05	0.458281	-0.017563	0.684865	-	-
Site 06	0.373158	-0.047579	0.557827	-	-
Site 07	0.332340	-0.075027	0.496707	-	-

It can be observed that there is no correlation between NDWI and the number of sandflies captured in the urban area. Regarding NDVI and number of sandflies we note the absence of correlation between the variables, i.e., the value of NDVI (with high or low) did not affect the number of vectors captured.

**Figure 1:**
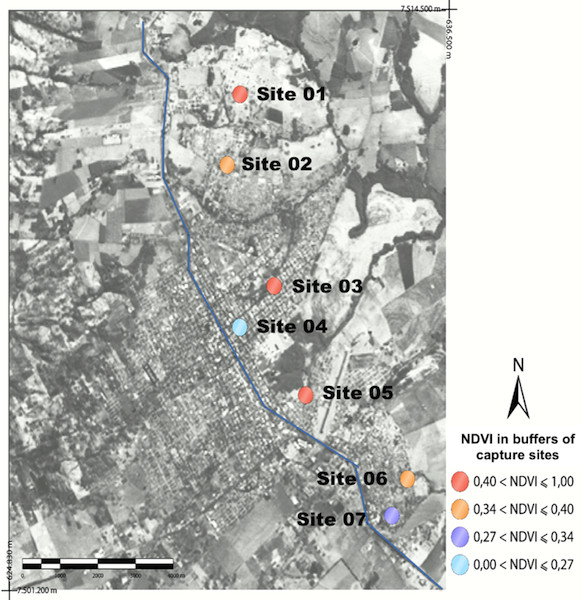
Landsat image with values of NDVI observed in the capture sites of the urban area of Ponta Porã, located on the right side of the map.

**Figure 2:**
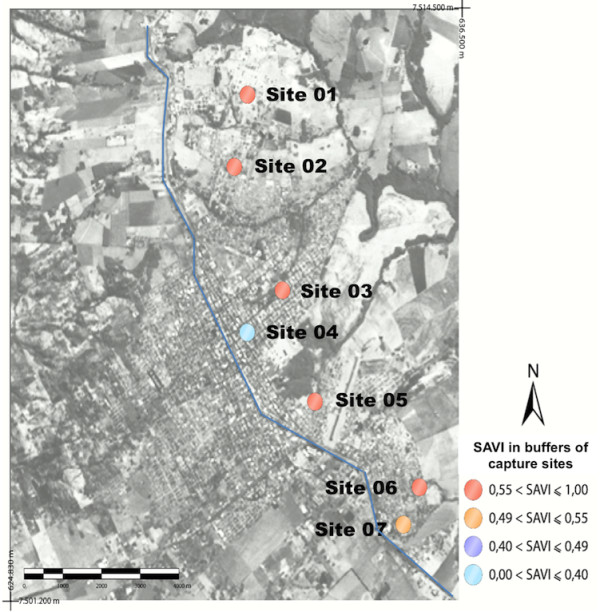
**Landsat image with the values of SAVI.** The figure shows, after correction, high values of vegetation in five of all seven sites studied.

**Figure 3:**
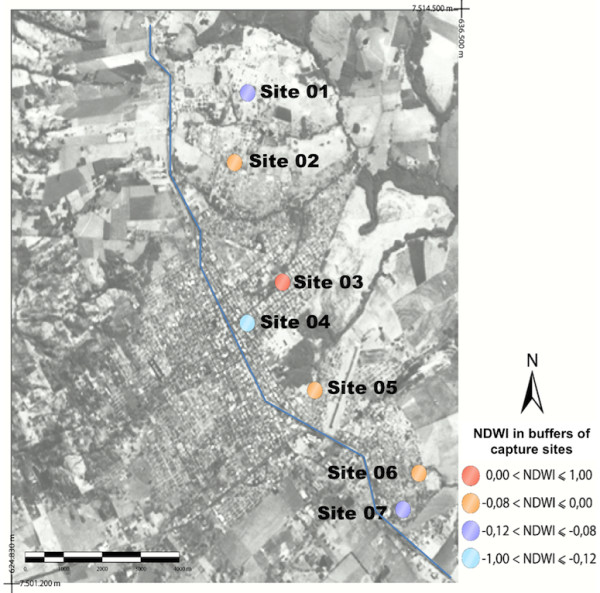
Landsat image with the values for the index of normalized difference by water (NDWI).

On the other hand, there was a negative correlation between NDVI and the number of canine cases, i.e., the lower the value of NDVI (vegetation or phytomass) the larger the number of canine cases existing in Ponta Porã. No correlation was seen between NDVI and the number of sandflies captured in the urban area, i.e. the value of NDVI (either high or low) did not affect the number of sandflies captured.The values for NDVI of the 7 capture sites can be observed in Figure 1.The correction of these values in different capture sites was performed using the vegetation index adjusted to soil background (SAVI), as can be seen in Figure 2.The values for NDWI of the 7 capture sites were showed in Figure 3.In Figure 1 it can be seen that, by the NDVI analysis, sites 2 and 6 presented median vegetation indexes (0.34 and 0.40). In Figure 2, after using SAVI, they came to present higher values. Site 3 was the one that showed the highest value of NDWI (the largest quantity of water).

Median values of water were obtained in sites 2, 5 and 6, corresponding to moderate moisture content. In site 4 minimum values of vegetation and moisture were found by the three indices (NDVI, NDWI and SAVI). In this site there was a concentration of cases of human and canine VL, besides the capture of *Lutzomyia longipalpis*.

## Discussion

The survey conducted in Ponta Porã suggests that the dynamics of the species population studied is similar to that reported by Barretto [20] and Aguiar *et al*. [21] in studies in which captures with light traps were found to have the advantage of offering a greater number of males. As possible hypotheses, there is also the fact that male hatching occurs before the females or the fact that the traps are installed next to the breeding sites. The fact that there are pets in the peridomicile also raises the possibility that males are being attracted to kairomones released by vertebrate hosts existing in ecotopes (dogs, chickens, human beings). There may have been attraction to traps when monitoring females for mating, since male sandflies are known to form “lekking” aggregates for the purpose of mating [22,23].

The occurrence and predominance of *Lu. longipalpis* in the intradomicile suggests its adaptation to artificial environments created by man, that serve as shelter for adult forms [24]. The presence of dogs in intradomicile and not in peridomicile was observed during the period of capture. The presence of dogs inside the houses is also considered an important factor, as they can act as a food source for females, serving as potential reservoirs of the disease, according to studies of Bigeli; Oliveira-Júnior; Teles [25].

In this study a negative correlation between NDVI and reported canine cases was observed. The concentration occurred in the same area where low vegetation index, which features a modified environment, was found. This finding is important from an epidemiological point of view, because the dogs act as the main domestic reservoirs of *Leishmania* in urban areas [26].

Margonari *et al*. [27] observed the occurrence of human and canine leishmaniasis in areas with presence of vegetation, which differs from that found in Ponta Porã.

Even though there is no correlation between the cases of human AVL, reports of LVC and the number of *Lu. longipalpis* collected, the presence of potential reservoirs and vectors specific for the agent in the urban area is important from the perspective of epidemiological surveillance. Considering that dogs are the main links in the epidemiological chain of disease in urban areas, and that many sandflies were collected in areas where the cases occurred, actions to control the disease must have priority [25,28-30].

There are no records of studies aiming to assess vegetation and its relation to the spatial distribution of vectors in areas of bordering countries. In Ponta Porã the analysis of vegetation cover by NDVI showed the presence of vegetation at high levels in three of the seven sites chosen. After using SAVI, five of the seven capture sites showed high levels of phytomass.

The central area of the city presents high values of NDVI, NDWI and SAVI, and also a wider diversity of phlebotomine species, which can be explained by the fact that Ponta Porã is surrounded by land with remnants of native forest, suggesting a higher degree of moisture in the environment, thus maintaining favorable conditions for vector development [9]. This result corroborates the findings of Fernandez *et al*. [31] that associated species to the presence of trees and bushes near capture sites in the urban area, which was also described by Cutolo, Camargo and Zuben [32] in studies developed in the southeastern region of Brazil.

In the State of Mato Grosso do Sul, Oliveira *et al.*[33] observed a wider diversity of species in forested areas in a study carried out in Campo Grande. Nunes *et al*. [34] also made this association in a survey conducted in Bonito. The association of vectors and areas with high vegetation index was also observed in a study performed in Campo Grande (MS) by Oliveira *et al.*[5]. Among the possible shelters for the sandflies described by Forattini [9], vegetation is suitable for maintaining breeding sites of winged and larval forms, presenting a wider diversity not only of plants that serve as a food source for males, but also vertebrate hosts acting as a food source for females.

NDWI is directly related to the water content in vegetation and soil moisture. It is an important fact since larval stages of sandflies require a humid environment, as noted by Amaral *et al.*[35]. There is no report in the literature as to an association of this index with the issue of leishmaniases.

The concentration of human and canine cases in a place where there was a greater capture of vectors was also observed by Prado *et al.*[29] in Montes Claros (MG); and Bigeli, Oliveira-Junior and Teles [25] in Palmas (TO). In Bahia, Carneiro *et al*. [28] observed low values of NDVI in sites where the numbers of human cases and the records of *Lu. longipalpis* were both high, which may suggest human activities on vegetation, corroborating what was observed in Ponta Porã.

The evaluation of moisture in the vegetation studied by NDWI is extremely important because it is directly linked to the development conditions of the insect’s immature form. The collection of *Lu. longipalpis* in all months of the year, associated with the presence of the sandfly in sites of low humidity, indicates that there is not a sole characteristic environment with specific conditions for the development and survival of these vectors.

## Conclusions

The finding of *Lu. longipalpis* in regions with little vegetation and humidity suggests that the species are adapted to different sorts of environmental conditions, demonstrating its close association with man and the environment he inhabits. The tourist feature of Ponta Porã reinforces its epidemiological importance as a vulnerable city. The geographical location, bordering Paraguay through dry borders, makes possible the existence of a corridor of vectors and infected dogs between the two countries.

## Competing interests

The authors declare that they have no competing interests.

## Authors’ contributions

AROA, RA conceived and designed the study; AROA, GC, APF, AR collected the data; AROA, BAKS analyzed data; AROA, BAKS, SMOA, MFCS wrote the manuscript. All authors read and approved the final version of the manuscript.
